# Comprehensive Cellular Senescence Evaluation to Aid Targeted Therapies

**DOI:** 10.34133/research.0576

**Published:** 2025-01-16

**Authors:** Xiaolan Zhou, Xiaofeng Zhu, Weixu Wang, Jing Wang, Haimei Wen, Yuqi Zhao, Jiayu Zhang, Qiushi Xu, Zhaozhao Zhao, Ting Ni

**Affiliations:** ^1^ State Key Laboratory of Genetic Engineering, National Clinical Research Center for Aging and Medicine, Huashan Hospital, Collaborative Innovation Center of Genetics and Development, Human Phenome Institute, Center for Evolutionary Biology, Shanghai Engineering Research Center of Industrial Microorganisms, School of Life Sciences, Fudan University, Shanghai 200438, China.; ^2^ Institute of Computational Biology, Helmholtz Center Munich, Munich, Germany.; ^3^ State Key Laboratory of Reproductive Regulation and Breeding of Grassland Livestock, Institutes of Biomedical Sciences, School of Life Sciences, Inner Mongolia University, Hohhot 010070, China.; ^4^ MOE Key Laboratory of Contemporary Anthropology, School of Life Sciences, Fudan University, Shanghai 200438, China.

## Abstract

Drug resistance to a single agent is common in cancer-targeted therapies, and rational drug combinations are a promising approach to overcome this challenge. Many Food and Drug Administration-approved drugs can induce cellular senescence, which possesses unique vulnerabilities and molecular signatures. However, there is limited analysis on the effect of the combination of cellular-senescence-inducing drugs and targeted therapy drugs. Here, we conducted a comprehensive evaluation of cellular senescence using 7 senescence-associated gene sets. We quantified the cellular senescence states of ~10,000 tumor samples from The Cancer Genome Atlas and examined their associations with targeted drug responses. Our analysis revealed that tumors with higher cellular senescence scores exhibited increased sensitivity to targeted drugs. As a proof of concept, we experimentally confirmed that etoposide-induced senescence sensitized lung cancer cells to 2 widely used targeted drugs, erlotinib and dasatinib. Furthermore, we identified multiple genes whose dependencies were associated with senescence status across ~1,000 cancer cell lines, suggesting that cellular senescence generates unique vulnerabilities for therapeutic exploitation. Our study provides a comprehensive overview of drug response related to cellular senescence and highlights the potential of combining senescence-inducing agents with targeted therapies to improve treatment outcomes in lung cancer, revealing novel applications of cellular senescence in targeted cancer therapies.

## Introduction

Targeted therapies disrupt specific signaling pathways that are critical for tumor growth [1]. Compared to chemotherapy, targeted therapies exhibit reduced toxicity and substantially improve treatment outcomes [2]. However, most single-agent targeted therapies can lead to resistance in advanced disease. Rational drug combinations have emerged as a promising strategy to overcome resistance; however, the vast number of potential combinations exceeds the scope of feasible clinical testing [3]. New combination strategies that can enhance the sensitivity of targeted therapies will have important implications for cancer treatment.

Cancer can be regarded as a condition that escapes the cellular fate of senescence [4]. Therefore, an innovative therapeutic approach known as the “one–two punch” has gained traction as a research hotspot [5]. This approach aims to first induce senescence in tumor cells, followed by senolytic treatment to target the anti-apoptotic pathways of senescent cells, thereby eliminating them [6]. Although many the US Food and Drug Administration (FDA)-approved drugs can induce cellular senescence, there are few successful clinical applications due to a range of complex issues related to the senescence microenvironment and the toxicity of senolytic compounds. It is worthwhile to explore more effective combination strategies between senescence-inducing drugs and targeted therapies.

A reliable evaluation of the senescence state in tumor samples is crucial for addressing the above issues. Single senescence markers, such as senescence-associated β-galactosidase activity or p16, are insufficient for accurately assessing the degree of senescence in tumor samples [7,8]. Several studies have identified and compiled numerous senescence-related genes, including CellAge [9], GenAge [10], aging/senescence-induced gene set (ASIG) [11], Aging Atlas [12], the senescence-associated secretory phenotype (SASP) pathway (R-HSA-2559582), SenMayo [13], and SenUp [14], which collectively provide a more comprehensive reflection of senescence states changes than single makers. These advancements allow for the evaluation of the senescence status of tumor samples based solely on gene expression profiles, which are readily accessible in public databases such as The Cancer Genome Atlas (TCGA).

In this study, we developed an enhanced strategy for evaluating cellular senescence scores (CSSs) based on 7 senescence-associated gene sets. We characterized the senescence-related molecular landscape of ~10,000 tumor samples from TCGA and identified 1,986 senescence-related genes in these tumors. Next, we examined the relationship between these senescence-related genes and the response of 251 anti-cancer drugs in cancer cell lines from the Genomics of Drug Sensitivity in Cancer (GDSC). Furthermore, we investigated the impact of senescence status on the response to 138 imputed drugs within TCGA. We validated that etoposide-induced senescence sensitized lung cancer cells to 2 targeted drugs, erlotinib and dasatinib. Finally, we explored the differential dependencies associated with senescence status across various cancer cell lines. Through these comprehensive analyses of cellular senescence status and drug response, we offered biological insights for the combination therapies involving senescence-inducing drugs and targeted drugs, uncovering novel applications of cellular senescence in cancer treatment.

## Results

### Development of the CSS metric to estimate cellular senescence status

**Fig. 1.**
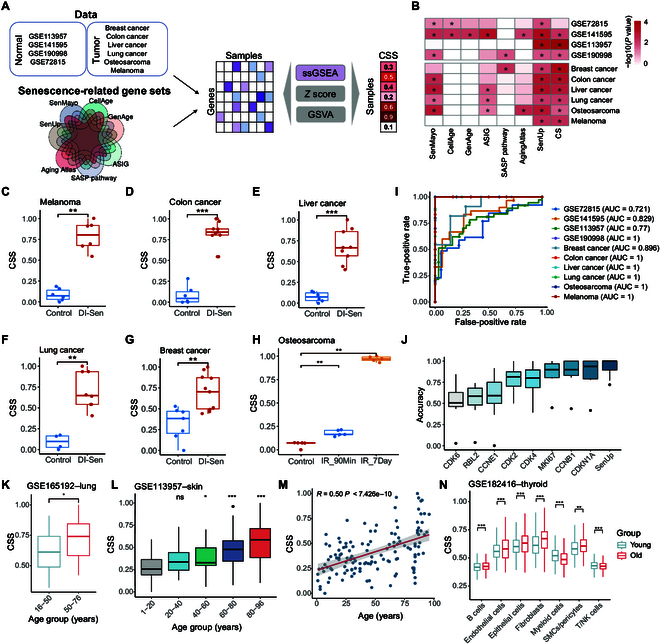
Development and evaluation of the cellular senescence score (CSS) metric. (A) Workflow for estimating senescence status from RNA sequencing (RNA-seq) data. The CSS is derived from the single-sample gene set enrichment analysis (ssGSEA) score, based on senescence-related genes from the SenUp gene set. SASP, senescence-associated secretory phenotype; GSVA, gene set variation analysis; ASIG, aging/senescence-induced gene set. (B) The senescence status of 10 public RNA-seq datasets was evaluated using the ssGSEA score derived from 7 different gene sets, alongside the previously reported cellular senescence (CS)-score method. The Wilcoxon rank-sum test was employed to assess the differences between senescent and nonsenescent samples. (C to G) Comparison of senescence scores based on the SenUp gene set between the control group (blue) and the drug-induced senescence (DI-Sen) group (red) across 5 cancer cell line datasets. The Wilcoxon rank-sum test was employed to assess the differences. (H) Senescence scores of osteosarcoma samples irradiated with 20 Gy, harvested at either 90 min (IR_90Min, blue) or 7 d (IR_7Day, orange). (I) Receiver operating characteristic (ROC) curves illustrating the performance of the CSS across 10 datasets with clearly defined senescence status labels. AUC, area under the dose–response curve. (J) Classification accuracies of the CSS compared to those of classic cell proliferation markers across 10 validation datasets. (K) Boxplots showing the comparison of CSS between different age groups in lung samples. The difference was estimated by the Student *t* test. (L) The distribution of CSSs across different age groups in human dermal fibroblast samples. The significance was tested (Student *t* test) for each age group in comparison to the 1 to 20 age group. (M) Scatterplot showing the Pearson correlation between CSS and age in human dermal fibroblast samples. (N) Boxplots showing the comparison of CSS between young and old groups across various cell types in normal thyroid tissue. SMCs, smooth muscle cells; NK, natural killer. The difference was estimated by the Student *t* test. For all panels, * indicates *P* < 0.05, ** indicates *P* < 0.01, *** indicates *P* < 0.001, and ns indicates not significant.

To quantify cellular senescence status using widely available gene expression profiles in tumor samples, we first collected 7 classic senescence-associated gene sets from various databases, including CellAge [9], GenAge [10], ASIG [11], Aging Atlas [12], SASP pathway (R-HSA-2559582), SenMayo [13], and SenUp [14]. Additionally, we collected 10 independent transcriptome datasets with known senescence statuses for validation (Fig. 1A). Subsequently, we employed 3 different algorithms (single-sample gene set enrichment analysis [ssGSEA], *Z* score, and gene set variation analysis [GSVA]) to calculate a score that reflects the relative abundance of senescent cells in each sample. Our results indicated that ssGSEA scores based on senescence-related genes from the SenUp gene set accurately distinguished samples with a high senescence status from those with a low status across all 10 datasets (Fig. 1B to G and Fig. S1F). For instance, osteosarcoma samples irradiated with 20 Gy and harvested either 90 min or 7 d later showed a significantly higher CSS than untreated samples (Fig. 1H). Furthermore, ssGSEA scores derived from the SenUp gene set outperformed those from other databases, demonstrating greater accuracy compared to scores calculated by *Z* score and GSVA (Fig. S1A to E). Consequently, we designated ssGSEA scores based on senescence-related genes from the SenUp gene set as the CSS for downstream analyses. We also evaluated the recently reported cellular senescence (CS)-score, a quantification metric for cellular senescence [15], across these 10 datasets and found that it could distinguish senescence status in 9 of the datasets (Fig. 1B). Our CSS metric (area under the curve [AUC]: 0.92) slightly outperformed the CS-score method (AUC: 0.90) in classifying senescence-high and senescence-low groups (Fig. 1I and Fig. S1D). Moreover, CSS also surpassed traditional cell cycle and senescence markers, such as *CDK2*, *CDK4*, *CCNB1*, and *CDKN1A* (Fig. 1J).

Senescent cells accumulate in various tissues during aging, which can result in enhanced tissue senescent cell burden [16]. We calculated the CSS of normal tissue samples in the Genome-Tissue Expression Project (GTEx) across different age groups. The results exhibited a significant increase in CSS with age in most tissues (Fig. S1H). This trend was further supported by RNA sequencing (RNA-seq) data from lung samples, which also demonstrated a significant increase in CSS with age (Fig. 1K). A significant positive correlation between CSS and age in human dermal fibroblast samples was also observed (Fig. 1L and M). Additionally, 10 patients with Hutchinson–Gilford progeria syndrome, a premature aging disease, exhibited higher CSSs compared to age-matched normal samples (Fig. S1G). In normal thyroid tissues profiled by single-cell RNA sequencing (scRNA-seq), we found that the CSS was higher in the old group compared to that in the young group across most cell types (Fig. 1N). Furthermore, tobacco smoking generates oxidative stress that can cause DNA damage and lead to stress-induced cellular senescence [17,18]. In squamous cell lung carcinoma, the CSS of current smokers were higher than that of ex-smokers, but this difference was not statistically significant (Fig. S1I). This may be attributed to the fact that bulk RNA-seq of tumor samples reflects the gene expression of multiple cell types. To further investigate, we downloaded high-throughput scRNA-seq data from primary bronchoalveolar lavage cells of current smokers and never smokers to compare CSS distributions across various cell types. We found that the CSS is higher in the smoking group compared to that in the nonsmoking group in most cell types (Fig. S1J). There results demonstrate that our CSS effectively evaluates senescence status from gene expression profiles in both normal and tumor samples.

### Global landscape of cellular senescence status across multiple cancer types

**Fig. 2.**
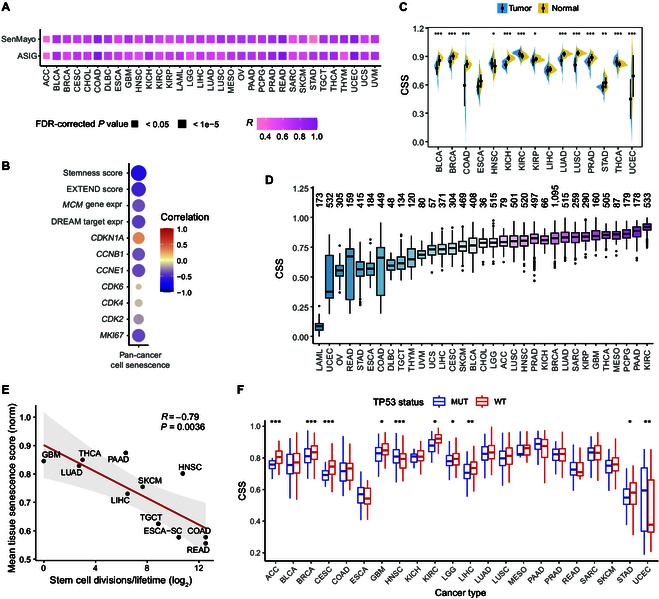
The landscape of senescence heterogeneity in The Cancer Genome Atlas (TCGA). (A) Pearson correlations between the CSS and senescence scores estimated using 2 other gene sets, SenMayo and ASIG, across various cancer types. FDR, false discovery rate. (B) Pearson correlation analysis of CSS with stemness score, telomerase activity (EXpression-based Telomerase ENzymatic activity Detection [EXTEND] score), and several commonly used markers of cell proliferation and senescence. expr, expression. (C) Comparison of senescence scores between primary tumors (blue) and adjacent normal solid tissues (yellow). A total of 15 cancer types, each containing at least 10 tumor samples and paired normal samples, were analyzed. (D) Boxplots showing the comparison of CSS across different cancer types, with the number of analyzed samples for each cancer type indicated above the boxplot. (E) Pearson correlation of mean CSS with estimates of stem cell division across different cancer types. (F) Comparison of CSS between tumors with *TP53* mutations and those without *TP53* mutations across various cancer types. MUT, mutant; WT, wild type.

We subsequently analyzed the cellular senescence status of ~10,000 tumor samples across 33 cancer types from the TCGA dataset. The CSS was highly consistent with the ssGSEA scores based on genes from SenMayo and ASIG (Fig. 2A). By examining the relationship between senescence status and other various molecular features, we found that the CSS exhibited a significant negative correlation with stemness score, telomerase activity, the expression of DNA replication-related gene families (e.g., *MCM2* to *MCM7*), and several proliferation genes (e.g., *CCNB1*) (Fig. 2B and Fig. S2A and C). Additionally, the CSS showed a strong positive correlation with senescence markers such as *CDKN1A* and SASP factors (Fig. 2B and Fig. S2B), indicating the robustness of the CSS in capturing cellular senescence signals in TCGA patient samples.

We then compared the senescence status of primary tumor samples with that of matched normal tissues and found that primary tumor samples exhibited lower CSSs but greater variability than normal samples across cancer types (Fig. 2C), consistent with previous findings that escape from senescence promotes tumor growth [4]. Furthermore, we observed that different tumor tissues displayed varying senescence scores (Fig. 2D). Tumor samples from pancreatic adenocarcinoma (PAAD), thyroid carcinoma (THCA), and glioblastoma multiforme (GBM) exhibited relatively higher CSSs, while samples from rectum adenocarcinoma (READ), colon adenocarcinoma (CODA), and esophageal carcinoma (ESCA) had lower CSSs. This phenomenon may be explained by the innate proliferative capacity of different tissues. Indeed, the stem cell division rates of different tissues showed a strong negative correlation with CSS (Fig. 2E, Cor = −0.79, *P* value = 0.0036).

The p53/p21/DREAM (dimerization partner, RB-like, E2F, and multi-vulval class B) axis regulates numerous cell-cycle-associated genes that are pivotal in cellular senescence [19]. By examining the relationship between CSS and the p53/p21/DREAM axis, we found that tumors proficient in *TP53* or exhibiting higher *CDKN1A* (which encodes p21) expression, along with those containing components of the DREAM complex, had elevated senescence scores across many cancer types such as stomach adenocarcinoma (STAD), breast invasive carcinoma (BRCA), lung adenocarcinoma (LUAD), and liver hepatocellular carcinoma (LIHC) (Fig. 2F and Fig. S2D and E). Notably, only 54 out of 525 genes in SenUp are classified as high-confidence p53 target genes (Fig. S2H) [20]. We also evaluated the association of cellular senescence with other mutations in TCGA. The most significantly associated mutated driver gene was *BRAF*, observed across multiple cancer types, including THCA and skin cutaneous melanoma (SKCM) (Fig. S2F and G). This finding is consistent with previous research, which has demonstrated that sustained *BRAF* mutation, predominantly V600E, in human melanocytes induces cellular senescence [21]. These results provide a comprehensive overview of the senescence status of tumors across different cancer types.

### Characterization of senescence-associated molecular signatures in cancer

**Fig. 3.**
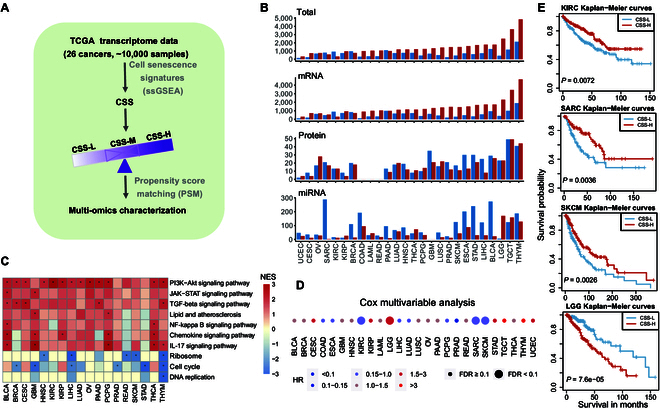
Characterization of multi-omics signatures associated with senescence status across different cancer types in TCGA. (A) Workflow for classifying senescence status. (B) Number of multidimensional senescence-associated molecular signatures in the CSS-low (CSS-L; blue) and CSS-high (CSS-H; red) groups from TCGA. Red represents signatures that are up-regulated in the CSS-H group, while blue indicates those up-regulated in the CSS-L group. CSS-M, CSS-medium; mRNA, messenger RNA; miRNA, microRNA. (C) Heatmap illustrating significantly dysregulated genes between the CSS-H and CSS-L groups, enriched in various senescence-associated pathways. PI3K, phosphoinositide 3-kinase; JAK, Janus kinase; STAT, signal transducer and activator of transcription protein; TGF-beta, transforming growth factor-β; NF, nuclear factor; IL-17, interleukin 17; NES, normalized enrichment score. (D) Relationship between cellular senescence status and overall survival times of patients, as calculated by the Cox proportional hazards model. HR, hazard ratio. (E) Kaplan–Meier curves for the overall survival of patients in the CSS-L and CSS-H groups across kidney renal clear cell carcinoma (KIRC), sarcoma (SARC), skin cutaneous melanoma (SKCM), and brain lower-grade glioma (LGG).

Next, we aimed to identify molecular signatures closely associated with senescence status in cancers. In total, 26 cancer types containing at least 100 tumor samples were included in the analysis. Tumor samples were classified into CSS-high (CSS-H), CSS-medium (CSS-M), and CSS-low (CSS-L) groups according to the distribution of score tertiles in each cancer type (Fig. 3A). To minimize the potential clinical confounders (e.g., sex, race, tumor purity, pathologic stage, and histological type), we employed the widely used algorithm propensity score matching [22,23].

By comparing 3 different molecular signatures including 20,501 messenger RNAs (mRNAs), 200 proteins, and 743 microRNAs (miRNAs) between the CSS-H and CSS-L groups, we identified a number of molecular signatures significantly associated with the cellular senescence status of tumors across various cancer types (Fig. 3B). The number of significantly altered molecular signatures varied greatly across different cancer types, exemplified by mRNA expression, which ranged from 403 genes in uterine corpus endometrial carcinoma (UCEC) to 6,577 genes in thymoma (THYM) (Fig. 3B). Protein expression alterations ranged from 6 in UCEC to 98 in testicular germ cell tumors (TGCT), while miRNA expression changed varied from 27 in kidney renal clear cell carcinoma (KIRC) to 315 in THYM. Moreover, the genes significantly altered at the mRNA layer in each cancer were enriched in senescence-associated pathways (Fig. 3C and Fig. S3A and B). Gene set enrichment analysis identified several key cellular senescence pathways, such as the interleukin 12 signaling pathway and phosphoinositide 3-kinase (PI3K)–Akt signaling pathway, which were significantly up-regulated in the CSS-H group. In contrast, several cell-growth-related pathways such as cell cycle and DNA replication were significantly suppressed in the CSS-H group, further supporting the effectiveness of our CSS in capturing cellular senescence signals in TCGA.

We examined the correlations between cellular senescence status classification and patients’ overall survival times using the Cox proportional hazards model. Our analysis revealed significant heterogeneity in these correlations across different cancer types. Notably, patients with high senescence scores exhibited a significantly better prognosis in KIRC, sarcoma (SARC), and SKCM. In contrast, patients with high senescence scores had a significantly worse prognosis in lower-grade glioma (LGG) (Fig. 3D and E). These findings support the context-dependent onco-suppressive and protumorigenic effects of senescence in pathophysiology [24,25]. Overall, our results indicate that patients with varying senescence statuses possess distinct molecular signatures.

### Integrative analysis of senescence-associated molecular signatures on drug response in GDSC

**Fig. 4.**
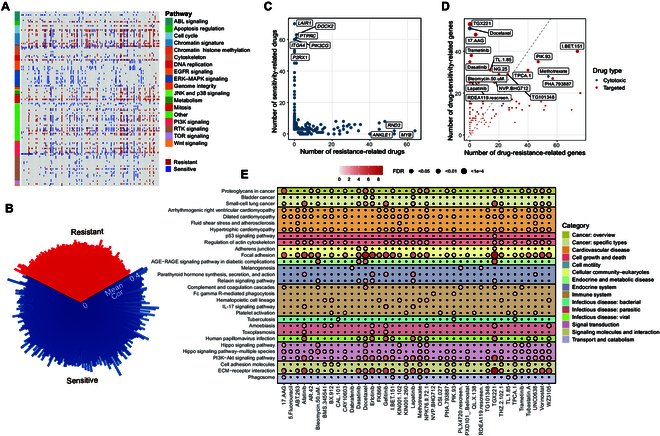
Associations between senescence-associated signatures and drug response in Genomics of Drug Sensitivity in Cancer (GDSC). (A) Correlations between the expression of senescence-associated genes and drug responses. Drug resistant (red) indicates that the expression of senescence-associated genes is associated with increased AUC values of drugs during senescence. Drug sensitive (blue) indicates that the expression of senescence-associated genes is associated with decreased AUC values of drugs during senescence. A smaller AUC represents greater sensitivity of tumor cells to a drug. EGFR, epidermal growth factor receptor; ERK, extracellular signal-regulated kinase; MAPK, mitogen-activated protein kinase; JNK, c-Jun N-terminal kinase; RTK, receptor tyrosine kinase; TOR, target of rapamycin. (B) Absolute mean Spearman’s correlation coefficients between the expression of senescence-associated genes and the response of their significantly correlated drugs. (C) Scatterplot showing the number of resistance-related and sensitivity-related drugs for each senescence-associated gene. (D) Scatterplot showing the number of resistance-related and sensitivity-related genes for each drug. (E) Kyoto Encyclopedia of Genes and Genomes (KEGG) pathway enrichment analysis of genes associated with different drugs. The pathways significantly enriched in at least 3 drugs are shown. AGE, advanced glycation end product; RAGE, receptor for advanced glycation end product; ECM, extracellular matrix.

To comprehensively explore the potential effects of senescence-associated signatures in cancer therapy, we focused on 1,986 differentially expressed cellular-senescence-associated genes between CSS-L and CSS-H groups (see Methods). We calculated the correlation between the expression levels of these genes and the area under the dose–response curve (AUC) of 251 anti-cancer drugs from the GDSC database across various cancer cell lines. These drugs can be broadly categorized into 2 groups: 232 targeted agents (including senolytic agents) and 19 cytotoxic agents. The targeted drugs target multiple key biological pathways such as the cell cycle, epidermal growth factor receptor (EGFR) signaling, PI3K signaling, receptor tyrosine kinase signaling, and the p53 pathway. We identified 229 senescence-associated genes significantly associated with the response of 128 drugs (including 122 targeted drugs) in at least 3 cancer types (|Cor| > 0.3, false-discovery-rate [FDR]-corrected *P* value <0.05; Fig. 4A). Notably, the majority of these 229 senescence-associated genes were associated with drug sensitization (Fig. 4B). For instance, *LAIR1* was up-regulated in CSS-H tumors across 6 cancer types, and its expression was associated with the sensitization of 70 anti-cancer drugs (Fig. 4C). Similarly, PIK3CG was up-regulated in CSS-H tumors in 11 cancer types, and its expression was correlated with the sensitization of 60 anti-cancer drugs (Fig. 4C). Among these genes, only 7 were identified as p53 target genes.

In addition, both cytotoxic and targeted drugs were associated with numerous drug-sensitivity genes (Fig. 4D). For instance, the drug response of lapatinib was negatively associated with 27 genes that were up-regulated in CSS-H tumors. These drug-associated genes participated in various biological processes such as cell motility, the immune system pathway, and signal transduction (Fig. 4E). Genes associated with PIK-93, a PI3K signaling pathway inhibitor, were significantly enriched in the phagosome pathway, extracellular matrix–receptor interaction, and the PI3K–Akt signaling pathway. Notably, the enrichment of genes linked to targeted drugs that affect the same pathways was closely clustered, as seen with EGFR signaling-targeted drugs gefitinib, afatinib, lapatinib, and erlotinib (Fig. S4). Collectively, our results demonstrate extensive associations between senescence-associated molecular signatures and the drug response of targeted therapies, highlighting the potential impact of cellular senescence status on clinical therapeutics.

### Functional effects of cellular senescence status on drug response in TCGA

We next performed an integrative analysis to assess the associations between cellular senescence status and drug response in TCGA. In total, 21 cancer types containing at least 30 tumor samples with imputed drug response information were used for analysis [26]. The imputed response data of 138 drugs (including 120 targeted drugs) of TCGA samples were obtained from a previous work that fitted a logical ridge regression model on the IC_50_ values of cancer cell lines [26]. Previous research on hypoxia and autophagy has yielded significant biological insights based on these imputed data [23,27].

**Fig. 5.**
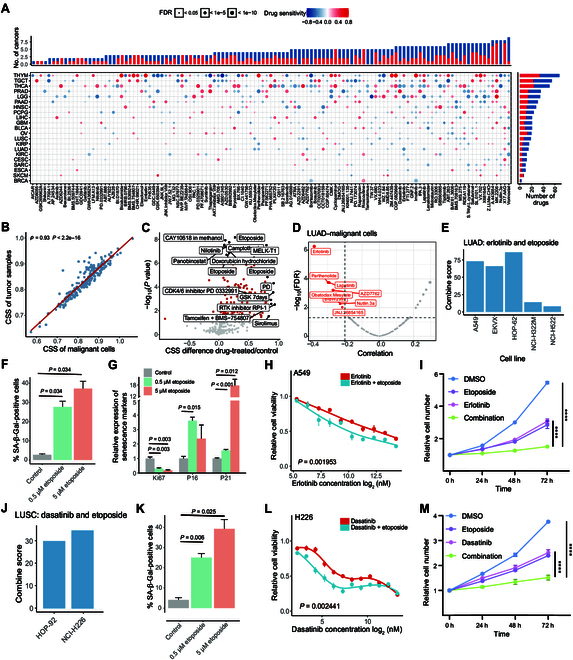
Effects of cellular senescence on targeted drug response in TCGA. (A) Spearman’s correlation between CSSs and drug responses in tumor samples across various cancer types (red indicates positive correlation, suggesting drug resistance; blue indicates negative correlation, suggesting drug sensitivity). The upper bar plot summarizes the number of cancer types with significant correlations for each drug, while the right bar summarizes the number of drugs with significant correlations for each cancer type. (B) Spearman’s correlation between the senescence status of malignant cells, as imputed by ENIGMA (dEcoNvolutIon based on reGularized Matrix completion), and the senescence status of tumor samples in lung adenocarcinoma (LUAD). (C) Volcano plot depicting differential CSS for each drug in the drug-treated group versus the control group. The *X* axis represents the difference of mean CSS between the drug-treated and untreated groups. The *Y* axis represents −log_10_ *P* value calculated using the Student *t* test for the mean CSS of the 2 groups. Red points indicate *P* value <0.05. (D) Scatterplot showing the correlation between the drug response of each drug and the senescence status of malignant cells imputed by ENIGMA in LUAD. Red points indicate drugs whose imputed response were negatively correlated with CCS among tumors. (E) The combination of erlotinib and etoposide in vitro yielded positive ComboScores across 5 LUAD cell lines, indicating enhanced activity compared to additive activity in vitro. (F) Quantification of senescence-associated β-galactosidase activity (SA-β-Gal)-positive cells of the A549 cell line treated for 48 h with dimethyl sulfoxide (DMSO), 0.5 μM etoposide, or 5 μM etoposide (images shown in Fig. S6J). Data were obtained in biological triplicate and analyzed using a 2-sided Student *t* test. Bars represent mean ± SD. (G) Quantitative reverse transcription polymerase chain reaction (qRT-PCR) analysis of the expression levels of 3 senescence-related marker genes in A549 cells. (H) Dose–response curves showing the mean cell viability of erlotinib under etoposide-induced and noninduced conditions in the LUAD cell line A549. Cell viability was normalized to the level of cells treated with DMSO. Data were analyzed using a paired Wilcoxon rank-sum test. (I) Quantitative analysis of the cell proliferation rate was performed using the Cell Counting Kit-8 (CCK-8) assay at different time points following the treatment of A549 cells. Treatments are as follows: DMSO, 1 μM etoposide, 80 μM erlotinib, and 1 μM etoposide + 80 μM erlotinib (combination). Error bars represent mean ± SD. The difference was estimated using the Student *t* test. (J) The combination of dasatinib and etoposide in vitro yielded positive ComboScores across 2 lung squamous cell carcinoma (LUSC) cell lines. (K) Quantification of SA-β-Gal-positive cells for the H226 cell line treated for 48 h with DMSO, 0.5 μM etoposide, or 5 μM etoposide (images shown in Fig. S7H). (L) Dose–response curves showing the mean cell viability of dasatinib under etoposide-induced and noninduced conditions in the LUSC cell line H226. (M) Quantitative analysis of the cell proliferation rate was evaluated using the CCK-8 assay at different time points following the treatment of H226 cells. Treatments are as follows: DMSO, 1 μM etoposide, 0.2 μM dasatinib, and 1 μM etoposide + 0.2 μM dasatinib. Error bars represent mean ± SD. The difference was estimated using the Student *t* test.

We calculated the Spearman’s correlation between CSS and the imputed drug response in TCGA. Our results indicated that the number of drugs associated with senescence status ranged from 3 in BRCA to 77 in THYM (|Cor| > 0.2, FDR-corrected *P* value <0.05; Fig. 5A). Interestingly, tumor samples with high CSSs exhibited increased sensitivity to many targeted drugs (Fig. 5A and Fig. S5A), aligning with trends observed in GDSC data (Fig. 4A and B). As anticipated, tumor samples with high CSSs were more sensitive to senolytic drugs such as ABT-263 [28] (Fig. S5B). We also found that CSS-H tumor samples demonstrated greater sensitivity to multiple inhibitors of the EGFR, PI3K, and ABL signaling pathways. This finding is consistent with previous study showing that EpiSen-high cells (identified as AXL-CLDN4+ cells; EpiSen: epithelial senescence) were more sensitive to EGFR and PI3K inhibitors in 2 head and neck squamous cell carcinoma cell lines [29]. Collectively, these results suggest that cellular senescence may enhance the sensitivity of tumor cells to specific clinical targeted therapies, providing a comprehensive overview of drug response related to cellular senescence status across various cancer types.

### Cellular senescence sensitizes drug response to targeted agents in vitro

Lung cancer is the leading cause of cancer-related deaths worldwide [30] and is the most common diagnosed cancer type in both males and females in 2023 [31]. Therefore, we further examined the drug response sensitized by a cellular senescence inducer in lung cancer. Given that the bulk gene expression profile of a tumor sample reflects a mixture of expression profiles from various cell types, we employed deconvolution methods, including our recently developed ENIGMA (dEcoNvolutIon based on reGularized Matrix completion) [32] and widely used BayesPrism [33], to estimate the cell-type-specific expression profile for each lung cancer sample, including LUAD and lung squamous cell carcinoma (LUSC). We found that malignant cells were the most abundance cell type in the tumor samples (Figs. S6A and S7A), and the senescence status of malignant cells showed a significant positive correlation with the senescence status of tumor samples across lung cancer and other cancer types, including ovarian cancer (OV), THCA, and LIHC (Cor > 0.9, *P* value < 0.001; Fig. 5B and Figs. S6B and C and S7B). This suggests that the CSS of tumors could reflect the senescence status of malignant cells in these cancer types.

Cellular senescence can be induced by many drugs with lower concentrations, leading to apoptosis at high concentrations [34,35]. We collected 66 transcriptomic studies on drug treatment effects according to a previous study [36], including over 200 targeted therapy and chemotherapy drugs. Indeed, the majority of drug-treated samples exhibited higher CSSs compared to untreated samples, particularly in those treated with etoposide (Fig. 5C).

In LUAD tumors, we found that senescence status exhibited the most significant correlation with the imputed erlotinib response based on both bulk tumor samples and deconvoluted malignant cells (Fig. 5D and Fig. S6D and E). This finding was further validated using data from the Cancer Therapeutics Response Portal version 2 (CTRPv2) dataset (Fig. S6F). The A Large Matrix of Anti Neoplastic Agent Combinations (NCI-ALMANAC) database contains the therapeutic activity results of over 5,000 pairs of FDA-approved drugs against a panel of NCI-60 (60 well-characterized human tumor cell lines) [37]. In NCI-ALMANAC, the combination of erlotinib and etoposide demonstrated a greater growth-inhibitory effect than additive activity, as indicated by a positive ComboScore in 5 LUAD cell lines (Fig. 5E). Additive activity is defined as the sum of the pharmacological effects of each drug in the combination [38]. Etoposide, a topoisomerase II inhibitor, induces DNA stress and cellular senescence in cancer cell lines [39,40]. Indeed, WI-38 fibroblasts [41] treated with 50 μM etoposide for 1, 2, and 7 d exhibited a higher CSS compared to untreated samples (Fig. S6G). Similarly, A549 lung cancer cells [42] treated with 2 μM etoposide also showed an increased CSS relative to that of untreated samples (Fig. S6H). Erlotinib, an EGFR-targeting tyrosine kinase inhibitor, was approved as a second-line treatment for non-small-cell lung cancer in 2004 [43]. In the NCI-ALMANAC, A549 cells treated with the combination of erlotinib and either 0.5 or 5 μM etoposide for 2 d demonstrated improved therapeutic effects compared to those treated with erlotinib alone (Fig. S6I). We experimentally confirmed the activation of senescence in A549 and H1299 lung cancer cells following treatment with 0.5 or 5 μM etoposide (Fig. 5F and G and Figs. S6J and L to N). We conducted drug sensitivity assays for erlotinib in A549 and H1299 cells under conditions of etoposide-induced cellular senescence compared to control conditions. We found that both LUAD cell lines exhibited increased sensitivity to erlotinib under etoposide-induced cellular senescence compared to control cells (Fig. 5H and Fig. S6O). Moreover, we found that the combination of etoposide and erlotinib led to a significant decrease in the proliferative capacity and colony formation of A549 and H1299 cells (Fig. 5I and Fig. S6K, 6P, and Q).

In LUSC tumors, we identified 12 drugs that exhibited a strong negative correlation with CSS such as dasatinib (Fig. S7C and D). Dasatinib and quercetin represent a classic senolytic combination that has been tested in several preclinical models such as those of the lungs, liver, and kidneys. However, dasatinib as a single agent has shown limited efficacy in eliminating tumor cells [44]. Our results indicated that the imputed dasatinib response and the IC_50_ values of dasatinib in the CTRPv2 database were significantly associated with the senescence status of tumors (Fig. S7E and F). In the NCI-ALMANAC, the combination of dasatinib and etoposide yielded a positive ComboScore in 2 LUSC cell lines (Fig. 5J), particularly in the H226 cell line (Fig. S7G). We experimentally confirmed that senescence was induced in H226 cells following treatment with 0.5 or 5 μM etoposide (Fig. 5K and Fig. S7H and I). Drug sensitivity assays showed that the LUSC cell line H226 exhibited increased sensitivity to dasatinib under etoposide-induced cellular senescence conditions (Fig. 5L). Furthermore, the combination of etoposide and dasatinib resulted in a significant decrease in the proliferative capacity and colony formation of H226 cells (Fig. 5M and Fig. S7J). Together, our results suggest that combining senescence-inducing agents (such as etoposide) with targeted therapies may enhance therapeutic efficacy in lung cancer.

### Differential dependencies associated with senescence status in CCLE

**Fig. 6.**
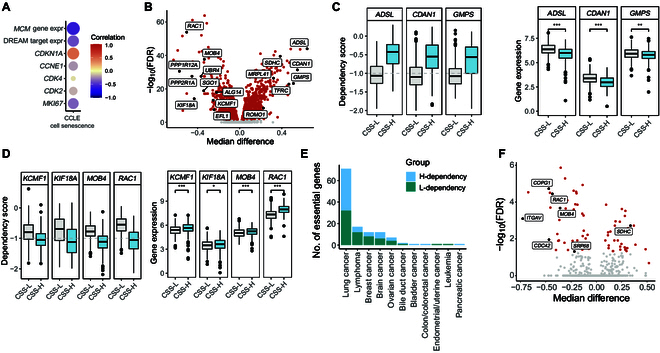
Differential dependencies associated with senescence status in the Cancer Cell Line Encyclopedia (CCLE). (A) Pearson correlation analysis between CSSs and various commonly used cell proliferation and senescence markers among cancer cell lines. The mini-chromosome replication maintenance (MCM) protein complex genes refer to the *MCM2* to *MCM7* gene family. (B) Volcano plot depicting differential gene dependency between CSS-L and CSS-H cell lines. The *X* axis represents the median gene effect score difference between the CSS-H and CSS-L groups. The *Y* axis represents the −log_10_ *P* value calculated using the Wilcoxon rank-sum test for the means of 2 groups’ gene effect scores. Red points indicate significantly different dependencies. (C) Boxplots displaying the dependency scores (left) and expression levels (right) of 3 genes (*ADSL*, *CDAN1*, and *GMPS*) that exhibit strong dependency in the CSS-L group. The threshold for dependency, as defined by dependency map, is indicated by the gray dashed line (−1). (D) Boxplots showing the dependency scores (left) and expression levels (right) of 4 genes (*KCMF1*, *KIF18A*, *MOB4*, and *RAC1*) that demonstrate increased dependency in the CSS-H group. (E) The number of genes showing differential dependency between CSS-L and CSS-H cell lines across different cancer types. H-dependency indicates increased dependency in CSS-H cell lines compared to that in CSS-L cell lines. (F) Volcano plot depicting differential gene dependency between the CSS-L and CSS-H cell lines in lung cancer.

To explore senescence-associated differential dependencies and uncover vulnerabilities in senescent cells that may benefit targeted therapies, we performed an integrative analysis using data from the Cancer Cell Line Encyclopedia (CCLE), which includes large-scale CRISPR-based genetic manipulation and gene expression data from ~1,000 cancer cell lines [45,46]. We first quantified the CSS for those cell lines. The CSS of cell lines showed a significant negative correlation with the expression of *MCM* family genes [47] and proliferation-associated genes (*CDK2*, *CDK4*, *MKI67*, and *CCNE1*)*.* It showed a positive correlation between CSS and the expression of senescence marker gene *CDKN1A* (Fig. 6A).

Next, we classified the cancer cell lines into 3 groups with different senescence statuses based on CSS and searched for genes with differential dependencies between the CSS-L and CSS-H cell lines. We identified 17 genes that were selectively essential to the CSS-L groups (Fig. 6B), exemplified by genes *ADSL*, *CDAN1*, and *GMPS*. The expression levels of *ADSL*, *CDAN1*, and *GMPS* were significantly higher in the CSS-L group compared to those in the CSS-H group (Fig. 6C). Guanosine monophosphate synthase, encoded by gene *GMPS*, catalyzes the synthesis of guanine monophosphate and plays roles in cell proliferation and DNA replication [48]. Knockdown of *GMPS* has been shown to reduce cell proliferation and promote cellular senescence and apoptosis in cervical [49] and liver cancer [50], underscoring its importance in cancer biology.

Interestingly, we also identified 30 genes with significantly increased dependency in CSS-H cell lines, exemplified by genes *KCMF1*, *KIF18A*, *MOB4*, and *RAC1*. The expression levels of those genes were significantly higher in the CSS-H group compared to that in the CSS-L group (Fig. 6D). Previous studies have reported that knockdown of *KIF18A* resulted in decreased cell proliferation and increased apoptosis in LUAD cells [51] and inhibited proliferation, migration, and invasion in esophageal cancer cells [52]. Additionally, deletion of *Rac1* led to reduced cell growth and increased apoptosis in primary mouse embryonic fibroblasts [53]. Genes with increased dependency in CSS-H cell lines were enriched in pathways related to oxidative phosphorylation, cell cycle, DNA replication, and various disease processes (Fig. S8C).

We also identified genes with differential dependency between senescence status groups for each cancer type separately, finding that lung cancer had the largest number of selectively essential genes in CSS-H versus CSS-L cell lines (Fig. 6E and F). These results reveal that the senescence status of cancer cells influences their dependency on specific genes, indicating that senescence may impact the effectiveness of targeted therapies.

## Discussion

Targeted therapies have reduced drug toxicity but have also led to increased drug resistance. Rational drug combinations represent a promising approach to enhance the treatment outcomes. However, the vast array of potential drug combinations exceeds the feasible scope of clinical testing. In addition, many FDA-approved drugs with lower drug concentrations can induce cellular senescence. Exploring effective drug combinations through the perspective of cellular senescence holds substantial clinical value. In this study, we defined a useful metric to quantify cellular senescence status. Utilizing this metric, we discovered that combining senescence-inducing drugs, such as etoposide, with anti-cancer targeted therapies can improve therapeutic efficacy.

In this study, we provided a comprehensive overview of senescence-related molecular signatures, including mRNA, miRNA, and protein expression. These signatures were enriched in various senescence-related and cancer-related pathways, such as cell cycle and IL-17 signaling. We provided a pan-cancer overview of cellular senescence status and its underlying gene dependencies, which could have potential clinical applications. Additionally, we provided a comprehensive landscape of drug response associated with cellular senescence across different cancer types. More importantly, we found several drug combinations that may be effective in lung cancer from the perspective of cellular senescence.

We initially explored the association between the expression of 1,986 senescence-related genes and drug response in the GDSC database. Our analysis revealed that the majority (88.2%) of these genes were associated with the sensitization of targeted therapy drugs, suggesting that cellular senescence may improve the effectiveness of targeted therapies in cancer treatment. Furthermore, we calculated the correlation between the CSS and the imputed drug response of 138 drugs to illustrate the overall impact of cellular senescence on anti-cancer targeted drug responses. Our results showed that most senescent tumor cells were associated with increased sensitivity to targeted therapy drugs, consistent with findings from the GDSC. As expected, tumor samples with high CSSs were more sensitive to senolytic drugs, such as ABT-263. Interestingly, these samples also exhibited increased sensitivity to multiple inhibitors of EGFR, PI3K, and other signaling pathways.

Senescent cells exhibit vulnerabilities and unique molecular signatures that may render them more susceptible to targeted therapies. Using the CTRPv2 dataset, the NCI-ALMANAC database, and in vitro experiments, we confirmed that etoposide-induced senescence sensitizes lung cancer cells to 2 target drugs: erlotinib and dasatinib (Fig. 5 and Figs. S6 and S7). These results indicate that the combination of senescence-inducing drugs and targeted therapies can lead to better therapy effect in lung cancer. However, it is important to note that this is a proof-of-concept study, involving 1 senescence inducer (etoposide) and 2 targeted drugs (erlotinib and dasatinib). With the comprehensive analysis and diverse drug data provided by this study, more attempts could be carried out by the research community to render more drug combinations for clinical application.

Several other challenges remain in our study that require further investigation. Firstly, our analysis relied on bulk RNA-seq tumor samples, and large-scale single-cell tumor databases do not provide drug response values. We employed deconvolution methods, ENIGMA and BayesPrism, to indirectly estimate the cell-type-specific expression profile for each tumor sample and investigated the correlation between the drug response and the CSS of malignant cells. With the development of single-cell profiling technology, future studies should account for tumor heterogeneity in anti-cancer drug therapies. Secondly, while etoposide is a widely used drug for inducing senescence, it may also trigger normal cellular senescence at lower doses. Most anti-cancer drugs can induce cellular senescence at lower concentrations; future research should focus on selecting drugs that specifically induce senescence in malignant cells. Finally, we experimentally demonstrated the phenomenon that senescent cells are more sensitive to certain drugs in lung cancer and used vulnerability to explain it, but the complex mechanisms underlying it remain underexplored in this work. Future investigations should delve into the genetic and biological pathways that enhance the sensitivity of senescent cells to targeted drugs through transcriptomic analysis and additional experiments. In summary, our study emphasizes the importance of considering cellular senescence in future targeted therapies and provides new rational drug combination strategies in targeted therapies.

## Methods

### Evaluating cellular senescence status from transcriptomic data

We collected 7 widely used senescence-related gene sets (CellAge, GenAge, ASIG, Aging Atlas, SASP pathway, SenMayo, and SenUp) to estimate CSS. Among these, the SenUp gene set contains 526 consistently overexpressed genes identified through a meta-analysis of 20 replicative senescence microarray datasets from the Gene Expression Omnibus (GEO) [14]. Additionally, this study also identified 734 consistently underexpressed genes, which we refer to as the SenDown gene set. Other gene sets were manually compiled from the literature. The CS-score is a recently reported metric that quantifies cell senescence levels, defined as the difference between the scores of the SenUp and SenDown gene sets.

To validate the performance of these 7 gene sets and CS-score using three different algorithms (ssGSEA, *Z* score, and GSVA), we downloaded 10 datasets with known senescence labels from GEO, including GSE158743 (osteosarcoma), GSE152699 (melanoma), the Cancer SENESCopedia database (breast cancer, colon cancer, liver cancer, and lung cancer), GSE113957, GSE141595, GSE190998, and GSE72815. We used log_2_(TPM + 1) transformed expression data for the following analyses: The R package GSVA was employed to implement the ssGSEA, *Z* score, and GSVA scoring methodologies, with the “method” parameter set to “ssgsea”, “zscore”, and “gsva”, respectively, while all other parameters remained at their default settings. The senescence scores derived from the SenUp gene set were consistent with the senescence status across the 10 datasets. Therefore, we refer to the ssGSEA scores based on the senescence-related genes from the SenUp gene set as the CSS.

Furthermore, we calculated the CSS of normal samples from the GTEx database and cells from GEO (GSE165192 and GSE182416), to validate the accumulation of senescent cells during human aging. The gene expression profiles and age data of GTEx normal samples were obtained from GTEx Portal (V8; https://www.gtexportal.org/home/downloads/adult-gtex). To investigate the impact of smoking on senescence status, we calculated the CSS of tumor samples in TCGA and downloaded patient clinical information, including gender, race, and smoking status, from the Genomic Data Commons data portal. Patients who were not smoking at the time of the interview were classified as nonsmokers, while others were categorized as smokers. We then compared the distribution of CSSs across different cancer types while controlling for gender and race (White). The gene expression profiles and smoking status of squamous cell lung carcinoma samples were obtained from GSE12428. Additionally, the scRNA-seq data and smoking status of bronchoalveolar lavage samples were collected from a previous study [54], which include 4 never smokers and 5 current smokers.

### Classification and multi-omics signature analysis across different cancer types in TCGA

Multi-omics data, including mRNA expression, protein expression, miRNA expression, somatic mutations, and clinical data (race, gender, pathologic stage, histological type, and overall survival times) from 33 tumor types, were downloaded from the Xena platform (https://xenabrowser.net/). Tumor purity data were downloaded from the TIMER portal (http://cistrome.org/TIMER/download.html) and the Zenodo repository (https://zenodo.org/record/253193) and subsequently integrated. The senescence scores for tumor samples from TCGA were calculated based on the SenUp gene set using the ssGSEA method.

We then classified these tumor samples based on the distribution of tertiles within each cancer type, designating the lowest and highest tertiles as CSS-L and CSS-H groups, respectively. Cancer types with at least 100 samples were included for comparison of molecular alterations. Cancer types containing at least 20 samples with overall survival times in the CSS-L and CSS-H groups were utilized for survival analysis. To address potential confounding factors such as gender, tumor stage, and histological type, we employed the matching weights method of the propensity score matching algorithm to balance the CSS-L and CSS-H groups, ensuring that the standardized difference was less than 0.1. The FDR-corrected *P* value for each cancer type was calculated using the Benjamini and Hochberg method. Significance for mRNA, protein, and miRNA molecular alterations was determined based on the following criteria: mRNA: |FC| > 2, FDR-corrected *P* value <0.05; protein: |difference| > 0.2, FDR-corrected *P* value <0.05; miRNA: |FC| > 1.5, FDR-corrected *P* value <0.05.

The R package clusterprofiler was utilized to perform Kyoto Encyclopedia of Genes and Genomes (KEGG)/Gene Ontology enrichment analysis with parameters minGSSize = 3 and maxGSSize = 500. Additionally, fgsea was employed to implement gene set enrichment analysis based on the KEGG pathway dataset downloaded from the R package KEGGREST, with parameters minSize = 3, maxSize = 500, and nperm = 10,000.

### Analysis of drug response

We focused on 1,986 genes with significantly altered mRNA and protein levels in at least 4 cancer types or with significantly altered mRNA or protein levels in at least 6 cancer types. The mRNA expression matrix and the AUC for 251 anti-cancer drugs from the GDSC across cancer cell lines were downloaded from GDSC1000 resources. We calculated the correction between the expression of senescence-associated genes and the drug AUC using Spearman’s correlation, with statistical significance defined as |Cor| > 0.3 and FDR-corrected *P* value <0.05.

To further assess the relationship between drug response and senescence status in TCGA, we downloaded the imputed drug response of TCGA samples from a previous research that fitted a logical ridge regression model to the IC_50_ values of a subset of cell lines from the GDSC cohort [26]. We analyzed the Spearman’s correlation between CSS and the imputed drug response of 138 drugs, with |Cor| > 0.2 and FDR-corrected *P* value <0.05 as thresholds for statistical significance.

As in previous studies, a negative correlation relationship was defined as drug-sensitive, while a positive correlation relationship indicated as drug-resistant [23]. For up-regulated genes in CSS-H tumors, a negative correlation relationship was defined as drug-sensitive and a positive correlation relationship as drug-resistant. Conversely, for down-regulated genes in CSS-H tumors, a positive correlation relationship was defined as drug-sensitive, and a negative correlation relationship indicated drug resistance.

### Cell culture

The human LUAD cell line A549, H1299, and human LUSC cell line H226 were cultured in Roswell Park Memorial Institute 1640 medium (Gibco) supplemented with 10% fetal bovine serum (Gemini) and 1% penicillin and streptomycin (penicillin–streptomycin solution, 100×, Beyotime) at 37 °C in 5% CO_2_ (v/v). To induce cellular senescence, A549, H1299, and H226 cells were treated with etoposide (Aladdin, E121713) at concentrations of 0.5 and 5 μM for 48 h.

### Cell proliferation assay

We purchased erlotinib-HCl (E129310) and dasatinib (D125110) from Aladdin. The effects of these drugs on cell proliferation were assessed using Cell Counting Kit-8 according to the vendor’s instructions (Dojindo). We plated 3,000 cells in each assay of 96-well plates. After 24 h, cells were treated with a range of drug concentrations prepared by serial dilution, along with 1 μM etoposide or dimethyl sulfoxide (5 replicates per condition). The plates were incubated at 37 °C and 5% CO_2_ (v/v) for different time periods. After the treatment, 20 μl of the Cell Counting Kit-8 reagent was added to the culture wells and incubated for an additional 2 h. The absorbance at 450 nm was recorded with Spark (TECAN). Relative viability was normalized to the untreated 5 replicates.

### Colony formation assay

To evaluate the colony formation ability of the lung cancer cell lines in response to various drug treatments, cells were cultured in 6-well plates at a density of 4,000 (A549 and H226) or 3,000 cells per well (H1299). Following this, the cells were cultured in the presence of different concentrations of drug combinations for 4 d. The culture medium was aspirated, and the cells were fixed using 4% paraformaldehyde and then stained with 0.2% crystal violet (Sangon Biotech) for 15 min. The wells were then washed with phosphate-buffered saline, and images were captured.

### Cell line dependencies

We obtained expression data and CRISPR gene effect scores from the DepMap portal (https://depmap.org/portal/download/all/; DepMap Public 22Q2). The senescence scores of cancer cell lines from the CCLE were calculated using the SenUp gene set with the ssGSEA method. We classified these cancer cell lines into 3 groups based on tertile distribution, designating the bottom tertile as the CSS-L group and the top tertile as the CSS-H group. Genes exhibiting significantly increased dependency in the CSS-H group were selected based on the following criteria: (a) The gene effect score in the CSS-H group is <−1. (b) The gene effect score in the CSS-L group is >−1. (c) The FDR-corrected *P* value is <0.05. Only cancer types with at least 30 samples were included in the analysis of cancer cell line-special essential genes.

### Statistics and reproducibility

The difference between 2 groups were assessed using the Wilcoxon rank-sum test, with *P* value <0.05 or FDR-corrected *P* value <0.05 being considered significant. * indicates *P* < 0.05, ** indicates *P* < 0.01, *** indicates *P* < 0.001, and ns indicates not significant. The associations between 2 vectors were analyzed using Spearman’s correlation, employing the rcorr() function in R.

## Data Availability

Validated public expression datasets were downloaded from GEO, including GSE158743, GSE152699, GSE113957, GSE141595, GSE190998, GSE72815, and the Cancer SENESCopedia. Expression data for CCLE and CRISPR gene effect scores were obtained from the DepMap portal (https://depmap.org/portal/download/all/; DepMap Public 22Q2). Multi-omics data, including mRNA expression, protein expression, miRNA expression, somatic mutations, and clinical data of TCGA tumor samples, were downloaded from the Xena platform (https://xenabrowser.net/). Tumor purity data were downloaded from the TIMER portal (http://cistrome.org/TIMER/download.html) and the Zenodo repository (https://zenodo.org/record/253193) and then integrated. The mRNA expression matrix, drug sensitivity data (AUC), and drug annotation information of GDSC were downloaded from the GDSC1000 resources (https://www.cancerrxgene.org/gdsc1000/GDSC1000_WebResources/Home.html). Drug sensitivity in GDSC is defined by 2 continuous metrics: IC_50_ and AUC, representing the half-maximal inhibitory concentration and the area under the dose–response curve, respectively. Lower IC_50_ or AUC values indicate greater drug sensitivity. The reference dataset for deconvolution is available in ArrayExpress under accession E-MTAB-6149 [55].
