# Supplementary figures and images for: Comprehensive Cellular Senescence Evaluation to Aid Targeted Therapies
Source: Research (Wash D C). 2025 Jan 16;8:0576. doi: 10.34133/research.0576 (PMC11735710; doi:10.34133/research.0576)

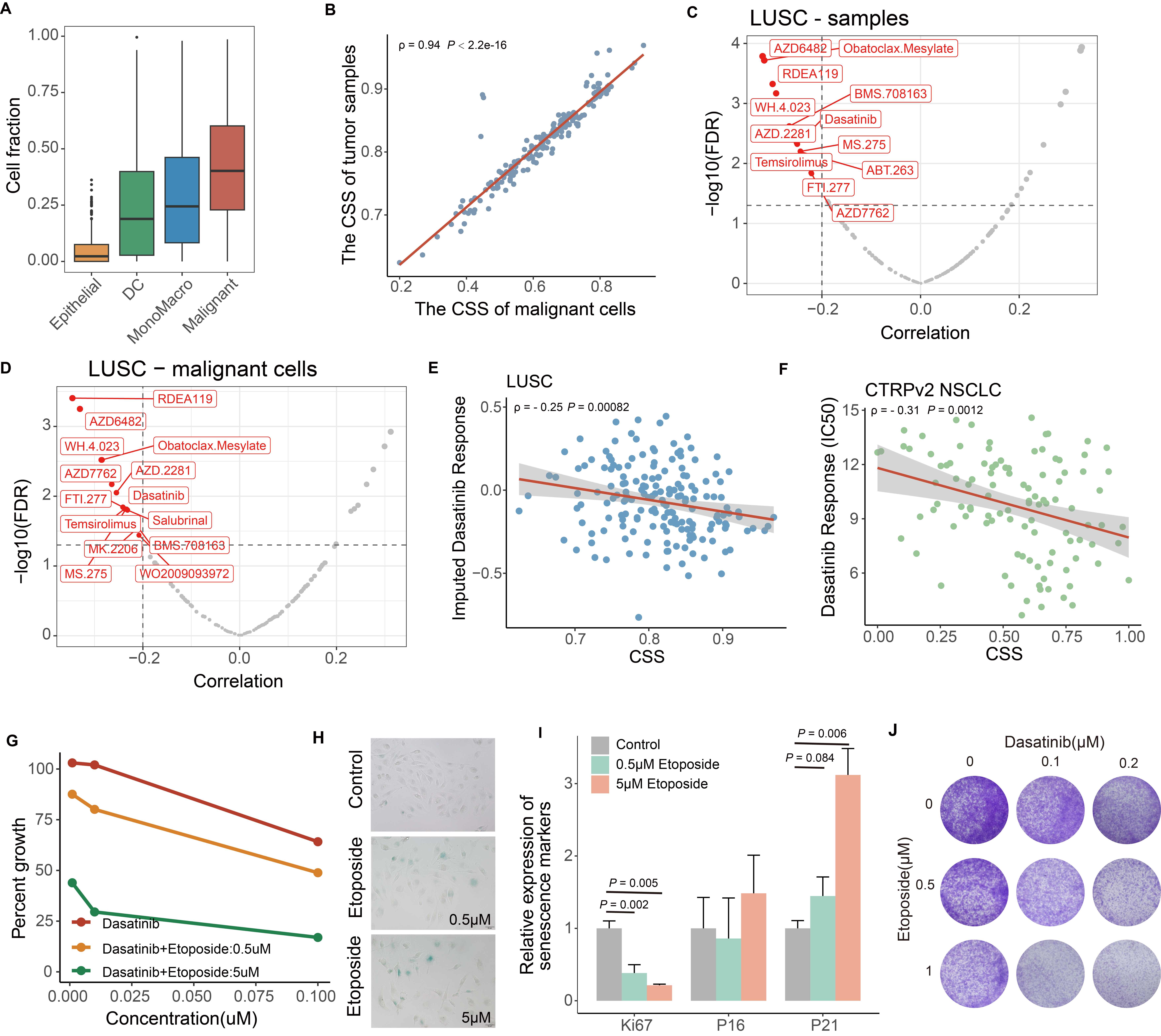

Supplement: Supplementary 1 — Figs. S1 to S8 [file research.0576.f1.zip › Fig.S7.png]
